# Dynamic microRNA Signatures as Biomarkers for Cardiac Ischemia and Remodeling

**DOI:** 10.3390/ijms27031488

**Published:** 2026-02-03

**Authors:** Macarena Rodríguez-Serrano, Elena Martín-García, Patricia Alonso-Andrés, Elisa Conde-Moreno, Héctor Pian, Javier del Moral-Salmoral, Nunzio Alcharani, Miriam Menacho-Román, Lorena Crespo-Toro, Miren Edurne Ramos-Muñoz, Carlos Zaragoza, Luis Miguel Rincón, María G. Barderas, María Laura García-Bermejo

**Affiliations:** 1 Biomarkers and Therapeutic Targets Group, Pathology Department, Ramón y Cajal Health Research Institute (IRYCIS), C/Carretera Colmenar Km 9,100, 28034 Madrid, Spain; e.makarena@hotmail.es (M.R.-S.); elena.marting01@gmail.com (E.M.-G.); patrialan22@gmail.com (P.A.-A.); elisa.condem@gmail.com (E.C.-M.); hector.pian@salud.madrid.org (H.P.); jdelmoralsalmoral@gmail.com (J.d.M.-S.); lorena.crespo.toro@gmail.com (L.C.-T.); edurneramosmunoz@gmail.com (M.E.R.-M.); 2 European Infrastructure for Translational Medicine (EATRIS), De Boelelaan 1118, 1081 HZ Amsterdam, The Netherlands; 3 Unidad de Investigación Cardiovascular, Departamento de Cardiología, Hospital Ramón y Cajal (IRYCIS), Universidad Francisco de Vitoria, 28223 Madrid, Spain; 4 Centro de Investigación Biomédica en Red de Enfermedades Cardiovasculares (CIBERCV), Instituto de Salud Carlos III, 37008 Salamanca, Spain; lmrincon@usal.es; 5 Clinical Biochemistry Department, Hospital Universitario Ramón y Cajal, IRYCIS, 28034 Madrid, Spain; 6 Department of Cardiology, University Hospital of Salamanca, Instituto de Investigación Biomédica de Salamanca (IBSAL), 37007 Salamanca, Spain; 7 Faculty of Medicine, University of Salamanca, 37007 Salamanca, Spain; 8 Department of Vascular Physiopathology, Hospital Nacional de Parapléjicos, SESCAM, 45071 Toledo, Spain; megonzalezb@sescam.jccm.es; 9 Department of Vascular Physiopathology, Hospital Nacional de Parapléjicos, IDISCAM, 45071 Toledo, Spain

**Keywords:** myocardial infarction, microRNA biomarkers, permanent coronary occlusion, cardiac remodeling, fibrosis, immune response, gene expression profiling

## Abstract

Myocardial infarction (MI) triggers complex pathological processes, including inflammation, hypoxia, and fibrotic remodeling. MicroRNAs (miRNAs) have emerged as promising biomarkers for cardiovascular injury; however, their expression dynamics along processes remain underexplored. We used an in vivo rat model of permanent coronary occlusion to study the molecular alterations associated with MI and its resolution in a temporal mode, including five experimental groups with five animals in each: sham, PO 24 h, PO 72 h, PO 7 d, PO 1 month. Histological analysis, serum biomarkers, and miRNA/gene expression profiles were analyzed in a time-dependent manner post-occlusion. Subsequent analysis revealed early depletion of selected circulating miRNAs (PO 24 h). Transient upregulation in cardiac tissue miRNAs, inflammatory and fibrotic gene expression (Fibronectin, Collagen, Vimentin, E-Cadherin) were observed at PO 72 h. These molecular alterations correlated with histological evidence of myocardial injury and repair. Taken together, our findings delineate the molecular timeline of MI progression and resolution and identify candidate miRNAs as sensitive and time-dependent indicators of myocardial stress, including miR-107, miR-122-5p and miR-221-3p. This integrative approach supports the use of miRNA signatures for noninvasive monitoring of cardiac injury and resolution and unveils potential therapeutic targets to reduce pathological remodeling.

## 1. Introduction

Cardiovascular diseases (CVDs) pose a significant threat to global health and represent the main cause of death in developed countries, with increasing prevalence in developing countries [1]. CVDs include pathologies in the circulatory system such as cardiac hypertrophy, myocardial infarction, heart failure and atherosclerosis. Conventional cardiovascular high-risk factors include arterial hypertension, diabetes, dyslipidemia or proinflammatory state [2].

Acute coronary syndrome (ACS), commonly known as myocardial infarction, is the most severe manifestation of coronary heart disease [3]. It is characterized by acute myocardial ischemia resulting from coronary artery plaque disruption and thrombosis-induced severe coronary artery stenosis or occlusion [4]. Symptoms of myocardial infarction include chest pain, shortness of breath, nausea, vomiting, abnormal heartbeat, stress or depression, among others [5].

ACS has a sudden onset of symptoms, with a preclinical phase in which the usual markers of target organ damage are useless. Currently, in medical practice, the diagnosis of myocardial infarction relies on a combination of ischemia symptoms, biomarkers indicating myocyte necrosis, electrocardiographic abnormalities, and cardiac troponin levels [6]. Regrettably, there is no specific set of early and precise biomarkers capable of predicting the occurrence or subsequent progression. However, recent studies, underscore the importance of circulating noncoding RNAs, particularly miRNAs, as valuable diagnostic and prognostic markers in acute coronary syndrome (ACS) management [7,8].

miRNAs are small noncoding RNAs involved in post-transcriptional gene regulation by inhibiting mRNA translation or promoting mRNA degradation [9,10]. miRNAs are secreted from cells and released into the circulation toward several tissues, free or into microvesicles, where they are stable and protected from RNase activity [11]. Some studies have investigated the key role of circulating RNAs in different pathologies and their development, including those affecting the cardiovascular system [12,13].

Myocardial infarction induces ischemic injury in myocardial cells [14], leading to metabolic consequences [15]. This injury triggers the loss of healthy mitochondria through the release of reactive oxygen species (ROS), hindering energy production and resulting in cardiomyocyte death [16].

The immune system is activated by cardiac injuries, which mediate the activation of cardiac fibrosis [17]. In myocardial infarction, an inflammatory environment arises with the activation of macrophages in the fibrous cap and subsequent proteolytic degradation of the matrix [18]. In addition, neutrophils [19] with specific time-dependent patterns and proportions [20], along with eosinophils, infiltrate the affected area. Eosinophils have been identified in the plasma of myocardial infarction patients, suggesting a potential protective role [21]. Some studies have shown that immune system cells are important for resolving post-MI injury, suggesting that neutrophils participate by polarizing macrophages toward a reparative phenotype [22]. Moreover, macrophages are pleiotropic cells that are associated with other cells to coordinate post-MI processes [23]. Marginal zone-B cells are also required to increase the mobilization of inflammatory monocytes to the ischemic myocardial tissue remodeling [24], suggesting that hypoxia promotes myocardial repair through the phenotypic transition of blood monocytes [25], suggesting the importance of the immune system in cardiac ischemia damage.

Previous work of our group identified miRNAs as useful biomarkers for MI diagnosis and prognosis, in a prospective study of MI patients hospitalized [8]. In the present study, we go further in the validation of these miRNAs and their association with tissue injury and remodeling, by means of a widely used experimental rat model of permanent occlusion reproducing the main features of MI in humans [26]. Our new findings reveal dynamic miRNA signatures able to monitor and predict the evolution of cardiac tissue injury after ischemia, easily detected in serum and consistent with our previous observations in patient serum samples, which could allow offering personalized management to MI patients.

## 2. Results

### 2.1. In Vivo Permanent Occlusion Rat Model Reproduces Heart Failure: Characterization of Tissue Alterations During Occlusion

**Figure 1:**
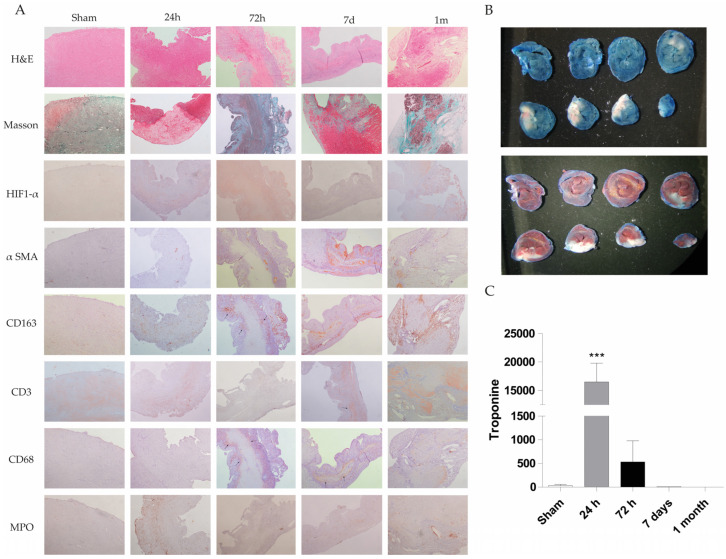
Histological, immunohistochemical and biochemical evaluation of cardiac injury in a rat permanent coronary occlusion model. (**A**) Representative histological sections of myocardial tissue at different time points (sham, 24 h, 72 h, 7 days and 1 month) were stained with H&E, Masson’s trichrome, and immunohistochemical markers (HIF-1α, α-SMA, CD163, CD3, CD68 and MPO) (200X magnification). Histological evaluation includes changes in tissue architecture, fiber disorganization, epicardial and endocardial cellularity, myocyte integrity, nuclear morphology, interstitial characteristics, and neovascularization. Notable findings include the onset of inflammatory infiltrates, necrosis and loss of myocytes (72 h–7 d), fibrosis, and increased vascularity in damaged areas. The table summarizes qualitative histopathological observations across time points, demonstrating progressive inflammation, immune cell infiltration, and tissue remodeling postinjury. (**B**) Evans blue and TTC staining were used to identify infarcted myocardial tissue in a representative rat. White areas indicate infarct zones, blue areas denote nonperfused tissue (Evans blue), and red areas indicate viable tissue (TTC). (**C**) Serum troponin I levels were quantified at different time points. Data represent mean ± SEM (n = 5 per group); *** *p* < 0.001 compared with the sham group.

The rat heart permanent occlusion model was used to reproduce myocardial infarction in humans. Briefly, in male Sprague-Dawley rats weighing 180–200 g, after anesthesia, permanent occlusion was induced by clamping the coronary descending aorta with a 7/0 silk suture through the 3rd and 4th intercostal spaces. Control animal group underwent the same surgical procedure without coronary clamping (n = 5 animals in each experimental group; sham, PO 24 h, PO 72 h, PO 7 d, PO 1 month; Total number equal 25 animals). A more detailed description of this model is provided in the Materials and Methods section. Paraffin-embedded heart tissue from these animals was stained with hematoxylin and eosin (H&E) to observe the tissue architecture and estimate the cardiac injury. Figure 1A shows H&E staining at 24 h, 72 h, 7 days and 1 month rat heart sections. Increased inflammatory cell infiltration associated with heart injury was observed, with a decrease in inflammatory cells and tissue transformation to a fibrotic state evident at 1 month. Masson staining results reveal collagen deposition at longer times (1 month) (blue staining) in the permanent occlusion heart section (Figure 1A).

Moreover, various molecular markers have been studied during the permanent occlusion process to understand the involvement of immune cells and other cells in the injury progression. These markers include CD68 (macrophage), CD163 (M2 macrophage), CD3 (T cell marker), HIF-1α (hypoxia-inducible factor), MPO (myeloperoxidase as neutrophil granulocyte) and α-SMA (α-smooth muscle actin for fibroblast activation).

Fiber disorganization was absent at 24 and 72 h post-permanent occlusion surgery. However, at 7 days, the tissues exhibited prominent infiltration, whereas at 1 month, the rats exhibited fiber disorganization and focal fibrosis. Specifically, there was an increase in cellularity at 24 h, 72 h, 7 days and 1 month, while the greatest increase was observed at 72 h. Endocardial tissue appeared normal at 24 h and 1 month, but focal affectation was noted at 72 h and 7 days after permanent occlusion. Cardiomyocytes appeared mature at 24 h, but they were replaced by inflammatory infiltrate at 72 h, 7 days and 1 month. Necrotic myocytes were identified at 72 h and 7 days, while fibrosis was observed at 1 month.

Nuclei exhibited low variability at 24 h with hyper-eosinophilia and focal pyknosis, whereas variability disappeared in focal areas at 72 h, 7 days and 1 month. Interstice changes were observed at 24 h with a slight increase in cellularity. At 72 h, inflammation with acute and chronic injury, necrosis, and hemorrhage were observed. Chronic inflammation was evident at 7 days and 1 month, with necrotic myocytes observed at 7 days. To resolve the injury process, new vascularity was increased at 72 h and highly increased at 7 days and 1 month in inflammatory areas.

In summary, the permanent occlusion model leads to myocardial tissue injury, including inflammatory response and neovascularization, validating this animal model for biomarker identification (Appendix A, Table A1).

In summary, these results indicate that permanent occlusion in the rat heart mimics acute myocardial infarction injury, with evidence of inflammatory infiltration in the tissue and increased serum troponin levels. After an infarction, the tissue undergoes transformation to a fibrotic state with collagen deposits.

To observe the infarct area, we employed Evans blue and TTC + Evans blue staining. White sections represent infarcted and ischemic areas on blue or red hearts stained with Evans blue or TTC + Evans blue, respectively (Figure 1B).

Quantification of the infarcted area as a percentage of the total left ventricular area in the shown Evans blue staining area has also estimated by two independent and blind pathologists, resulting in 46%.

Troponin levels were measured in the serum of a rat model of permanent occlusion to analyze cardiac function (Figure 1C). Troponin levels were significantly greater at 24 and 72 h post-permanent occlusion than in the sham control values, with a higher significance at 24 h. Troponin levels at 7 days and 1 month post-permanent occlusion were similar to or lower than those in the sham animals.

### 2.2. Serum miRNA Levels Decrease at 24 h in Permanent Occlusion Rats

A miRNA profile previously studied and published in patient sera by our group (Rincón LM et al. [8]) was determined by qRT-PCR in rat sera, including: miR-let7a-5p, miR-20a-5p, miR-21-5p, miR23a-3p, miR27b-3p, miR30b, miR93-5p, miR221-3p, miR210-3p, miR107, miR122-5p, miR144-3p and miR148-3p.

**Figure 2:**
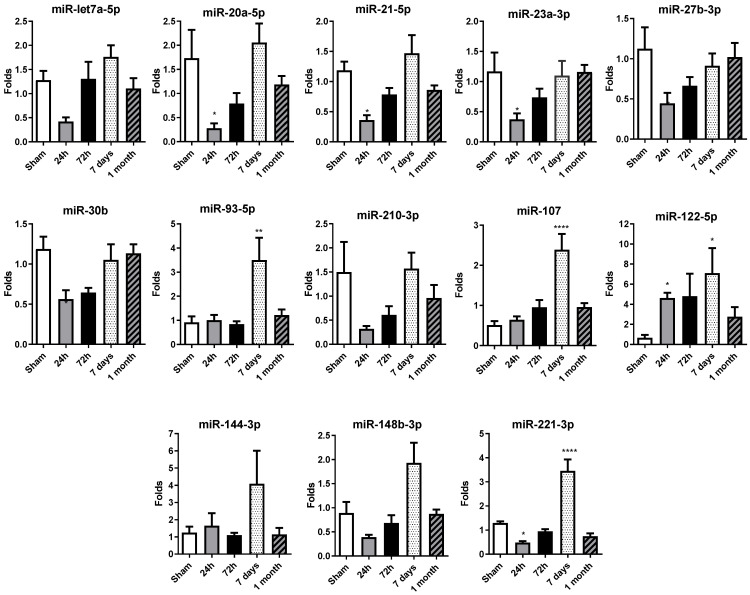
Temporal expression profile of selected circulating miRNAs in rat serum after permanent coronary occlusion. The expression of miR-let7a-5p, miR-20a-5p, miR-21-5p, miR-23a-3p, miR-27b-3p, miR-30b-5p, miR-93-5p, miR-107, miR-122-5p, miR-144-3p, miR-148b-3p, miR-210-3p and miR-221-3p was analyzed by RT-qPCR in serum samples collected at 24 h, 72 h, 7 days and 1 month after permanent coronary occlusion. Normalization was performed using miR-30c as an endogenous control. Data are expressed as the fold change relative to sham controls and presented as mean ± SEM (n = 5 per group). Statistical comparisons were conducted using one-way ANOVA followed by Dunnett’s post hoc test for normally distributed data or Kruskal–Wallis test for nonparametric data. * *p* < 0.05, ** *p* < 0.01, **** *p* < 0.001 vs. sham controls.

Serum miRNA levels decreased at 24 h, in comparison with the control sham group levels, in permanent occlusion rats, with significant alterations observed in the majority of miRNAs (Figure 2). It is important to highlight that miR-20a-5p, miR-21-5p, miR-23a-3p, miR-27b-3p, miR-30b and miR-221-3p exhibit a significant decrease in their expression levels at 24 h post-occlusion, suggesting an orchestrated early response to ischemic injury. The 72 h time point may represent a transitional phase with no differential expression in comparison with the sham or 24 h group, possibly contributing to the changes observed on day 7.

Interestingly, at 7 days, there was an overall increase in miRNA expression levels, with significant increases observed in miR-93-5p, miR-107, miR-122-5p and miR-221-3p. After one month, the miRNA expression profile was normalized, with most miRNA levels similar between the permanent occlusion and sham groups.

In summary, the marked decrease in circulating miRNA levels observed 24 h after permanent coronary occlusion represents a critical event in the early response to ischemic injury. The increase observed at 7 days would contribute most probably to the resolution phase, completed at 1 month, when miRNA levels resembled those of the sham animals.

### 2.3. miRNA Expression Increases at 72 h of Permanent Occlusion in Heart Tissue

**Figure 3:**
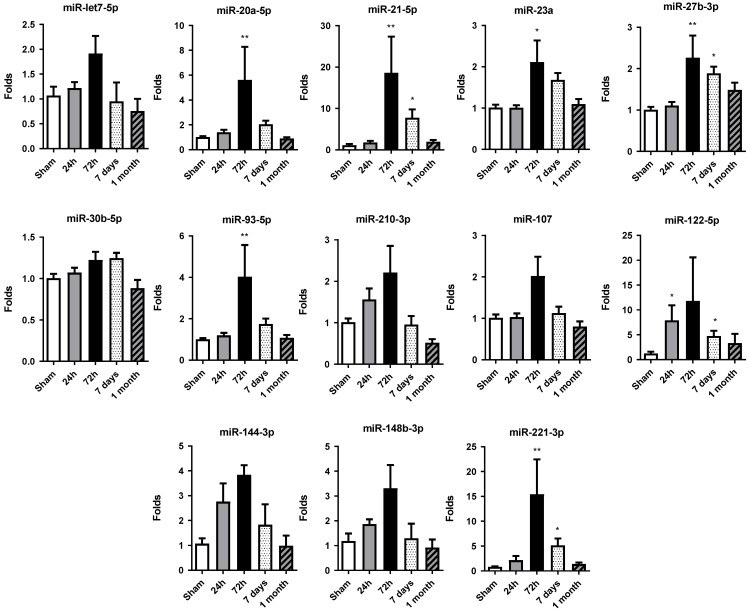
Temporal expression profile of selected miRNAs in rat cardiac tissue. The expression of miR-let7a-5p, miR-20a-5p, miR-21-5p, miR-23a-3p, miR-27b-3p, miR-30b-5p, miR-93-5p, miR-107, miR-122-5p, miR-144-3p, miR-148b-3p, miR-210-3p and miR-221-3p was quantified by RT-qPCR in heart tissue samples from rats subjected to permanent coronary occlusion. Time points analyzed were 24 h, 72 h, 7 days and 1 month post-occlusion. miR-30c was used as the endogenous reference for normalization. Data are expressed as fold change relative to sham controls and are presented as mean ± SEM (n = 5 per group). Statistical significance was assessed using one-way ANOVA followed by Dunnett’s post hoc test for normally distributed data or Kruskal–Wallis test for nonparametric data. * *p* < 0.05, ** *p* < 0.01 vs. sham controls.

Altered miRNA expression profiles were observed in rat hearts after permanent occlusion surgery, with the most significant changes occurring at 72 h post-occlusion (Figure 3). After that, the miRNA expression profile tended to be restored to the sham control group values.

At 24 h, only miR-122-5p showed significant increases in its level of expression compared to the sham group, albeit to a lesser extent at 72 h. At this time, miR-20a-5p, miR-21-5p, miR-23a-3p, miR-27b-3p, miR-93-5p and miR-221-3p were significantly increased with respect to the control group.

Moreover, in 7 days and 1 month, the general miRNA expression profile observed at 72 h tended to decrease. However, the expression at 7 days was still significantly increased compared to the sham group expression in miR-21-5p, miR-27b-3p, miR-122-5p and miR-221-3p. At 1 month, miRNA expression level was restored to levels detected in sham tissue, showing no significant differences among the miRNAs studied.

Overall, the increase in cardiac miRNA expression observed at 72 h post-occlusion reflects an adaptive response of the myocardium to sustain stress and damage and could be related to the increase in miRNA levels detected in serum.

### 2.4. Upregulation of Fibronectin, Collagen, E-Cadherin and Vimentin at 72 h Contrasts with Transient VEGF Downregulation After Permanent Coronary Occlusion

**Figure 4:**
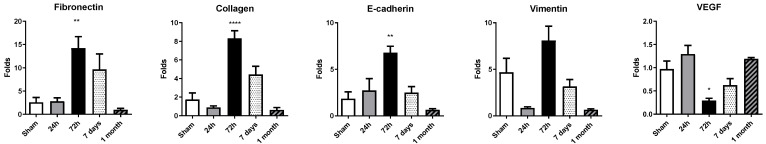
Expression of remodeling-related genes in rat cardiac tissue. Gene expression levels of fibronectin, collagen, E-cadherin, vimentin and VEGF were assessed by RT-qPCR in heart tissue collected at 24 h, 72 h, 7 days, and 1 month post-occlusion. Expression values were normalized to 28S rRNA and are presented as fold change relative to sham-operated controls. Data represent mean ± SEM (n = 5 per group). Statistical significance was determined by one-way ANOVA followed by Dunnett’s post hoc test for normally distributed data or Kruskal–Wallis test for nonparametric data. * *p* < 0.05, ** *p* < 0.01, **** *p* < 0.001 vs. sham controls.

Cardiac remodeling after myocardial infarction was mirrored by a dynamic regulation of fibrotic, epithelial-to-mesenchymal transition (EMT) and angiogenic genes (Figure 4). At 24 h, no statistically significant differences were found; a mild increase in expression was found for fibronectin, E-cadherin and VEGF, with a minor downregulation of collagen and vimentin. Pronounced upregulation of fibronectin, collagen, vimentin and E-cadherin was detected at 72 h after permanent coronary occlusion, indicating early activation of extracellular-matrix deposition and cytoskeletal reorganization. Among these transcripts, fibronectin displayed the greatest increase in expression compared with sham animals. By day 7 and 1 month, all four genes had downregulated their expression.

In contrast, VEGF exhibited a transient suppression at 72 h, followed by a nonsignificant upregulation at 7 days and 1 month.

These data delineate a temporal cascade in which an early response in gene expression at 72 h is followed by progressive normalization. These gene dynamics could synergize with miRNA dynamics, among others, for driving heart remodeling.

## 3. Discussion

In this study, we demonstrated that a permanent coronary-occlusion rat model mimicking heart failure provides insights into the temporal progression of cardiac tissue injury and resolution. In particular, miRNA profiles determined in tissue and serum could serve as biomarkers for heart failure development or abnormal remodeling in preclinical experimental settings, with promising implications in clinical practice.

In the permanent occlusion rat model, histological and molecular analyses revealed increased inflammatory cell infiltration at 24 and 72 h post-occlusion, transitioning to fibrosis after 1 month post-occlusion. This aligns with previous findings that inflammation precedes fibrosis in myocardial infarction models. The infarcted areas visualized by Evans blue and TTC staining, and echocardiographic functional studies confirmed the extent of ischemic damage, corroborating the observed increase in serum troponin levels, a marker of cardiac injury [27]. Masson’s trichrome staining further highlighted progressive collagen deposition, indicative of fibrotic tissue remodeling [28].

A detailed histological examination revealed dynamic changes in tissue architecture post-occlusion over time. Early stages (24–72 h) were characterized by increased cellularity and inflammation, including both acute and chronic injury markers. At 7 days and 1 month, there was evidence of chronic inflammation, fibrosis, and neovascularization. The presence of prominent infiltrates at 7 days, followed by fibrosis and fiber disorganization at 1 month, mirrors the stages of wound healing and tissue remodeling [29], strongly suggesting that the animal model used in this manuscript reproduces heart failure features and evolution in humans.

In this scenario, we explored the temporal dynamics of miRNA expression following permanent coronary occlusion in vivo and correlated these changes with key molecular and cellular events occurring during injury, inflammation, and tissue remodeling. The data presented here highlight that distinct profiles of miRNAs are regulated over time, most likely driving the shift in pathophysiological processes from the initial acute ischemic insult to the later reparative and fibrotic phases.

Numerous studies have reported that miRNA profiles are associated with cardiovascular diseases, particularly myocardial infarction. The expression of miR-18a-5p, miR-27a-3p, miR-423-5p, miR-30e-5p, miR-26b-5p, miR-233-3p and miR-16-5p is lower in acute heart failure patients than in healthy individuals [30]. Individual miRNAs such as miR-144-3p could also be considered new biomarkers for cardiac fibrosis progression after myocardial infarction [31]. In a mice model, PEMFs combined with adipose-derived stem cells upregulated miR-20a-5p expression, which was associated with reduced left ventricular dilatation and improved cardiac function postinfarction, suggesting that miR-20a-5p is a potential biomarker of ventricular remodeling [32]. On the contrary, miR-223 protects against hypertrophy and heart failure [33], and miR-19a/19b stimulate cardiac proliferation and regeneration after myocardial infarction [34].

In the present study, in the early stages (24–72 h) post-infarction, acute cell death, increased inflammation, and the onset of tissue hypoxia drive significant alterations in circulating and tissue miRNA expression. Specific miRNAs, such as miR-21-5p, miR-122-5p, and miR-221-3p and miR-27-3p, were significantly modulated during these early stages. The elevated presence of miR-122-5p in serum could potentially serve as a noninvasive biomarker of acute myocardial injury in this experimental setting, showing robust changes over time that correlate with the severity of tissue damage and inflammatory processes. Accordingly, with our findings, miR-146a and miR-21 have been proposed as predictive biomarkers of left ventricular remodeling after ST-elevation myocardial infarction in humans [13]. miR-155 can participate in neovascularization, and miR-21 is considered a key molecule in myocardial dysfunction. miR-122-5p or miR-223-3p are proposed as useful biomarkers of plaque instability also in patients [35].

Our findings also align with previous studies showing increased levels of miR-21-5p, miR-122-5p, and miR-320a-3p in the plasma of patients with cardiogenic shock [36]. Additionally, other studies have proposed the miR-122-5p/miR-133b ratio as a prognostic biomarker in acute myocardial infarction [37]. Similarly, miR-21-5p, which is known to be involved in processes such as inflammation, angiogenesis, and fibrotic remodeling [13], exhibited notable changes in both serum and tissue, reflecting the cardiac tissue-specific response as well as circulating signaling processes.

Our findings demonstrate that later time points (7 days–1 month) evidenced a transition from acute damage to tissue remodeling and fibrosis, characterized also by altered expression of miRNAs commonly associated with extracellular matrix organization and angiogenesis. The total or partial return of certain miRNAs to baseline levels at 1 month suggests that some of these molecular responses are transient and related to the active phases of tissue repair. Notably, miR-107, miR-122-5p and miR-221-3p were modulated in serum at these later stages, indicating their potential roles in guiding tissue regeneration or in reflecting ongoing vascularization and cell survival strategies.

On the other hand, we demonstrate that the increased expression of fibronectin, collagen, E-cadherin, and vimentin at 72 h post-occlusion correlates with the acute phase of tissue injury and cellular reprogramming. The expression of this gene then partially decreases at the largest stages. Therefore, a robust fibrotic response is observed early on, followed by a transition into longer-term remodeling, such as in the classical phases of infarct healing, where initial inflammation and extracellular matrix deposition transition into a more organized tissue structure [38].

The identification of miRNAs that consistently show altered expression patterns in the early phase of ischemic damage in experimental animal models could be of clinical interest. Early and accurate biomarkers are critical for timely diagnosis and intervention in patients with myocardial infarction. Our results revealed that miRNAs such as miR-21-5p, miR-122-5p, and miR-221-3p emerge as promising biomarker candidates. Each of these miRNAs displayed robust changes correlating with injury or remodeling phases in experimental settings. Specifically, early increases in miR-122-5p in serum samples may reveal the severity of tissue damage and the onset of adverse remodeling processes. As these miRNAs are easily detectable in circulation, as previously demonstrated by our group [9], they could potentially contribute to improving the current diagnostic standards that rely heavily on cardiac troponin measurements. For this purpose, future research should focus on validating these candidate miRNAs in independent and larger patient cohorts, deciphering their specific molecular targets, and evaluating their sensitivity and specificity compared with those established biomarkers.

Our findings also indicate that the temporal concordance between miRNA expression kinetics and remodeling-gene expression supports the presence of a highly regulated miRNA–mRNA connection that drives postinfarction healing in our permanent-occlusion model. The tissue upregulation of miR-21-5p, miR-20a-5p, miR-27b-3p, miR-93-5p and miR-221-3p at 72 h correlates with the maximal induction of fibronectin, collagen, vimentin and E-cadherin, genes that drive extracellular-matrix deposition and epithelial-to-mesenchymal transition.

Functional studies modulating the expression of the above-mentioned miRNAs could help confirm their role in regulating these remodeling pathways. In this regard, several of these miRNAs have documented pro-fibrotic activities; miR-21-5p regulates the PTEN-Akt signaling pathway [39,40], enhancing fibroblast activation, contributing to cardiac repair. Delivery strategies involving miR-21 mimic to specific heart cells have shown potential to reduce damage and improve heart function after injury [41]. Related to neovascularization, miR-93-5p is known as a translational repressor of VEGF [13]. This simultaneous increase provides a mechanistic explanation for the transient suppression of VEGF we detected at 72 h, implying that the injured myocardium prioritizes structural stabilization over angiogenesis during the acute phase.

Finally, our findings revealed a general decrease in circulating miRNAs at 24 h, followed by their enrichment in cardiac tissue at 72 h, suggesting rapid redistribution from blood to the damaged myocardium involving EV, among other mechanisms. After 72 h, both miRNA and gene levels decreased progressively (7 days to 1 month), indicating the transition from a pro-inflammatory/pro-fibrotic stage to a more organized remodeling stage. Moreover, the associated miRNA–mRNA dynamics not only could delineate the temporal course of cardiac repair but also could unveil a therapeutic interval of approximately 72 h in which targeting these miRNAs might attenuate adverse remodeling, in experimental settings.

## 4. Materials and Methods

### 4.1. Permanent Occlusion Rat Model

**Figure A1:**
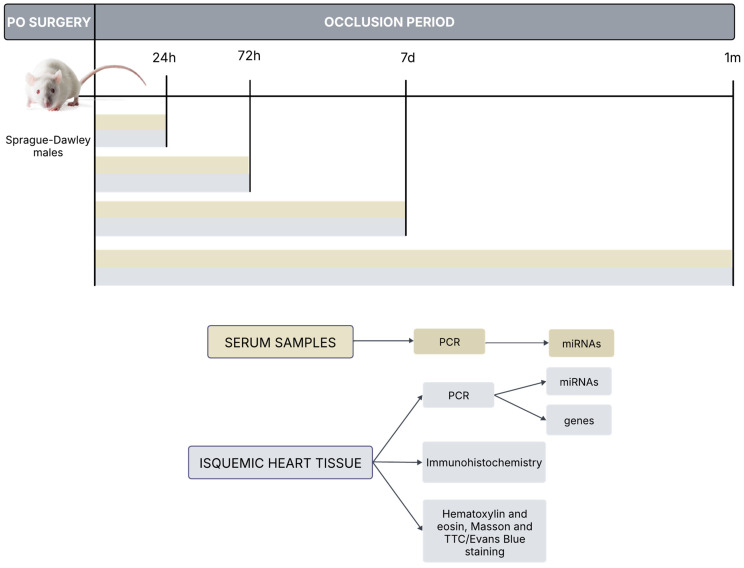
In vivo permanent coronary occlusion rat model: Experimental procedures.

In order to emulate a myocardial infarction, a widely used rat model of permanent occlusion of the coronary artery was established on 8–10-week-old male Sprague Dawley rats (Charles Rivers, Wilmington, MA, USA), with an average weight of 200 g, which was achieved by ligating the left descending (LDA) coronary artery, following international guidelines for MI experimental animal models [26]. Individual rats were housed with free access to water and fed with standard food *ad libitum*. Anesthesia was induced with 5% isoflurane and maintained at 2%, while body temperature was preserved with an electric blanket. Rats were intubated and connected to an automatic ventilator. Eyes were protected with a drop of Metozel (Omnivision, Santa Clara, CA, USA), and analgesic Tramadol-Adolonta (Grunenthal, Aachen, Germany) was subcutaneously administered (30 mg/kg). Additionally, 1% lidocaine (Hospira INC, Lake Forest, CA, USA) was subcutaneously injected into the chest incision area. Permanent occlusion was induced by clamping the coronary descending aorta with a 7/0 silk suture through the 3rd and 4th intercostal spaces. Control animal group underwent the same surgical procedure without coronary clamping (n = 5 animals in each experimental group; sham, PO 24 h, PO 72 h, PO 7 d, PO 1 month; Total number equal 25 animals) (Appendix A, Figure A1). MRS and LMR were aware of the group’s allocation.

The thoracic wall was closed using 4/0 silk suture, and the skin was closed with 3/0 silk suture. Ceftriaxone (Normon, Paris, France), an antibiotic and tramadol analgesic were administered to aid animal recovery. Blood was extracted via tail vein puncture at 24 h, 72 h, 7 days and 1 month. Rats were sacrificed at the same time, and hearts were harvested to carry out further experiments.

All animals were purchased from Charles Rivers Laboratories, France, and housed in the Hospital Universitario de la Paz animal facility, taking into account proper husbandry. All experimental animal approaches were approved by the Hospital la Paz ethical Committee in compliance with European and Spanish guidelines (PROEX 198/16).

### 4.2. Troponin Measure

Rat serum troponin was measured to assess acute myocardial damage [42]. The troponin determination was performed on the Abbott Alinity i analyzer, USA using a chemiluminescent microparticle immunoassay (CMIA), following the manufacturer’s instructions, at the Biochemistry Department, Ramón y Cajal Hospital.

Briefly, the cardiac troponin I present in the sample binds to the microparticles coated with antitroponin I antibodies. The mixture was washed. Acridinium-labeled antitroponin I antibody conjugate is added to create the reaction mixture and incubated. After a wash cycle, the preactivator and activator solutions are added.

The resulting chemiluminescent reaction was measured in relative light units (RLU), being a directly proportional reaction between the amount of cardiac troponin I and the RLU detected by the optical system.

### 4.3. Hematoxylin and Eosin, Evans Blue and TTC Staining

Hematoxylin and eosin staining (H&E) was conducted to visualize tissue structure, while TTC and Evans blue were used to determine the infarct tissue area in the permanent occlusion model.

For H&E staining, 3 µM tissue sections were first deparaffinized and hydrated in alcohol. Then, sections were washed in H_2_O, immersed for 2 min in hematoxylin, washed in H_2_O and submerged in eosin for 30 s. Following this, the tissue was dehydrated and mounted with Depex mounting media.

Evans blue staining was performed at the endpoint of the rat experiments by injecting Evans Blue Solution (50 μL of a 30 mg/mL solution in 0.9% normal saline, nonheparinized saline, or approximately 50 mg/kg) into the jugular vein catheter, followed by a small volume of heparinized saline to flush the line. Normal heart tissue appeared blue, whereas the infarcted area appeared white.

TTC staining [43] was performed after heart harvest, and Evans blue staining was performed. Hearts were washed with PBS, and the atrium and right ventricle were removed. The remaining tissue was frozen for 20 min at −20 °C, and then cut into 2 mm sections using a cryostat. These sections were immersed in preheated 1% TTC for 15 min at 37 °C in darkness, followed by washing 3 times in PBS. The infarcted area appeared white, while the other normal tissue appeared deep red. Quantification of the infarct size was estimated by two independent and blind expert pathologists and has been estimated as a percentage of necrotic area vs. risk area in the left ventricle.

### 4.4. Masson’s Staining

Masson’s trichrome staining was performed using Masson Trichrome Goldner kit (Bio-Optica Strumentazioni Scientifiche, Milano, Italy) to visualize collagen deposits. Tissue sections (3 µm) were first hydrated by immersion in absolute and 96° ethanol. Tissue was then immersed in 6 drops of reactive A and 6 drops of reactive B for 10 min. Subsequently, 10 drops of reactive C were added for 4 min, followed by washing in H_2_O. Sections were then incubated with 10 drops of reactive D for 4 min, repeating this step with reactive E. Excess staining was removed without washing, and reactive F was added for 5 min. After washing, the sections were dehydrated by rinsing in alcohol and mounted in Depex mounting media (Leica, Wetzlar, Germany). Masson’s staining resulted in collagen deposits appearing green in the connective tissue.

### 4.5. Immunohistochemistry Protocol

Heart rat tissues were processed and embedded in paraffin. Sections (3 µm) of paraffin-embedded tissues were hydrated prior to antigen retrieval in Dako Target Retrieval Solution, following the manufacturer’s instructions. Endogenous peroxidase activity was blocked by incubating the slides in Peroxidase-blocking solution (Dako, Santa Clara, CA, USA) for 30 min at room temperature. Slides were incubated with 1% BSA-PBS for 30 min at room temperature before primary antibody incubation. Primary antibodies, against CD68 (1:500, Abcam ab 125212, Cambridge, UK), CD3 (1:100, Abcam ab16669), CD163 (1:300, Abcam ab182422), myeloperoxidase (1:75, Thermo Scientific PAS16672, Waltham, MA, USA), alpha smooth muscle actin (α-SMA) (1:200, Abcam ab7817) and hypoxia inducible factor 1-alpha (HIF1-α) (1:20, Novus Biological NB 100–105) were incubated overnight at 4 °C (Table 1). EnVision + Dual Link secondary antibody (Dako) and DAB (Dako) solution were used for immunohistochemistry visualization. Finally, sections were dehydrated and mounted with Depex mounting media (Leica). Estimations have been performed in a random and blinded manner by 2 independent and expert observers.

### 4.6. RNA Isolation from Rat Serum and Heart Tissues

Serum was processed directly with miRNeasy Serum/Plasma Advanced Kit (Redwood City, CA, USA) following the manufacturer’s protocol, using 1 μg MS2 (Roche, Basel, Switzerland) as a carrier. Heart tissues from rats were sectioned to obtain slides of the infarcted area. The tissue was mechanically disaggregated (TissueLyser LT, Qiagen, Germantown, MD, USA) and lysed in TriPure Isolation Reagent. Total RNA was isolated following the manufacturer’s protocol. RNA was resuspended in 20 µL of nuclease-free water.

### 4.7. RT-qPCR for miRNAs and Genes from Serum and Heart Rat Tissues

RNA was quantified by Nanodrop 2000 (Thermo Scientific) and transcribed to cDNA (Qiagen) using at least 200 ng of RNA (tissues) as template for miRNAs determination. 4 µL of total RNA was used as a template from serum samples. UniSp2 Spike-in (Qiagen) added during RNA isolation served as the extraction control. miR-cel-39 was used as a reverse transcriptase efficiency control. An amount of 4 µL of diluted cDNA (1/11) was used as template for the master SYBR Green Kit (Exiqon, Woburn, MA, USA) and used for amplification with LNA-modified primers for increasing specificity, following the manufacturer’s instructions.

For genes, total RNA was transcribed to cDNA (Roche) using 2 µg of RNA as template, and 1 µL of cDNA was used for qPCR with the master SYBR Green kit (Qiagen). PCRs were run on a LightCycler 480 equipment (Roche). Triplicates were performed for each condition or sample and normalized to the 28S mRNA expression level for the genes and miR-30c for miRNA studies. These housekeeping genes or miRNAs were selected based on previous group results. Ct values were obtained using the 2nd derivative method (Light Cycler 480 Software 1.5, Roche). miRNA or gene expression was expressed as ΔCt = miRNA Ct/gene Ct expression − Ct miRNA/gene normalizer expression. qRT-PCR approaches have been performed in a random and blinded manner. Table 2 summarizes the primers used in this work.

### 4.8. Statistical Analysis

Data are presented as mean ± Standard Error of the Mean (SEM). The Levene test of homogeneity of variance was conducted, as well as one-way ANOVA followed by Dunnett’s post hoc test for normally distributed data or Kruskal–Wallis test for nonparametric data. A *p* < 0.05 was considered significant. Statistical analysis was performed using Statistical Package for the Social Sciences (SPSS) version 15.0.

The calculation of the sample size for each animal group is based on: (1) previous work with similar methodology, as observed in the bibliographic search, (2) the possibility of achieving statistical significance in a comparative analysis like the one proposed, based on the resource equation for this purpose, and (3) possible limitations of the model or the procedures employed.

### 4.9. Study Limitations

Despite the strengths of our study in delineating the temporal dynamics of miRNAs during myocardial infarction (MI), several limitations must be acknowledged. First, while the permanent coronary occlusion rat model effectively simulates the pathophysiology of human heart failure and remodeling, it does not fully replicate the clinical complexity of MI in humans, which often involves underlying comorbidities such as aging, diabetes, or hypertension, and typically undergoes reperfusion therapy (e.g., PCI). Second, our study utilized a relatively small sample size (n = 5 per group), which, while sufficient for identifying significant molecular trends, may limit the generalizability of the findings and the detection of subtle biomarker variations. Third, although we identified a strong correlation between miRNA signatures and the expression of remodeling genes like fibronectin and collagen, these findings remain associative; functional gain- or loss-of-function experiments are required to establish a direct causal link between these specific miRNAs and the observed histological outcomes. Finally, our molecular analysis was restricted to a pre-selected panel of miRNAs and genes based on previous literature; a broader, unbiased approach, such as small RNA sequencing, might reveal additional novel regulators of the post-infarction reparative phase.

## 5. Conclusions

The permanent coronary-occlusion rat model accurately reproduced the sequential events that follow myocardial infarction and resolution: acute necrosis, inflammatory infiltration, angiogenic signaling and fibrotic scar formation. Across our model, three microRNAs (miR-21-5p, miR-20a-5p, and miR-221-3p) displayed a robust, time-dependent pattern: an early decrease in the bloodstream during the first 24 h, followed by a pronounced increase in cardiac tissue at 72 h, parallel to peaks in fibronectin, collagen and E-cadherin expression. This coherent signature links circulating and local molecular responses to distinct phases of injury and remodeling.

Our data therefore identify miR-21-5p, miR-20a-5p, and miR-221-3p as sensitive, noninvasive biomarkers that can (i) detect early myocardial damage, (ii) track the shift from acute inflammation to scar formation and (iii) unveil potential novel therapeutic targets for intervention in cardiac remodeling, in experimental preclinical settings.

## Figures and Tables

**Table 1:** Antibodies, reference and dilution used in the immunohistochemistry protocol.

Antibody	Reference	Dilution
HIF1-α	NB 100–105	1:20
α-SMA	ab7817	1:200
CD163	ab182422	1:300
CD3	ab16669	1:100
CD68	ab125212	1:500
Myeloperoxidase	PAS16672	1:75

**Table 2:** miRNAs and genes primers.

miRNA	Primer Sequence	Gene	Primers Sequences
miR-21-5p	UAGCUUAUCAGACUGAUGUUGA	Fibronectin 1	F: GCCCTTACAGTTCCAAGTTCC
miR-23a-3p	AUCACAUUGCCAGGGAUUUCC		R: GCCTACATAACAACTCTTCTC
miR-122-5p	UGGAGUGUGACAAUGGUGUUUG	VEGFA	F: AAAAACGAAAGCGCAGAAA
miR-148b-3p	UCAGUGCAUCACAGAACUUUGU		R: TTTCTCCGCTCTGAACAAGG
miR-93-5p	CAAAGUGCUGUUCGUGCAGGUAG	Collagen 1α	F: GTGGAAACCTGATGTATGCT
miR-20a-5p	UAAAGUGCUUAUAGUGCAGGUAG		R: TGGTGATCATATTCTTCTGGG
miR-107	AGCAGCAUUGUACAGGGCUAUCA	CDH1	F: AGAAGCCATGACAAGTACCT
miR-30b-5p	UGUAAACAUCCUACACUCAGCU		R: ACAGATCCCTCAAAGACCTC
let-7a-5p	UGAGGUAGUAGGUUGUAUAGUU	Vimentin	F: CCTGCTCAATGTAAAGATGG
miR-144-3p	UACAGUAUAGAUGAUGUACU		R: GGTGTCAGTTGTTATGTGCT
miR-27b-3p	UUCACAGUGGCUAAGUUCUGC		
miR-221-3p	AGCUACAUUGUCUGCUGGGUUUC		
miR-210-3p	CUGUGCGUGUGACAGCGGCUGA		

## Data Availability

The original contributions presented in this study are included in the article. Further inquiries can be directed to the corresponding author.

## Abbreviations

The following abbreviations are used in this manuscript:

ACS	Acute Coronary Syndrome
α-SMA	Alpha-Smooth-Muscle Actin
AMPKα2	AMP-Activated Protein Kinase alpha-2 subunit
BMDMs	Bone-Marrow-Derived Macrophages
BSA	Bovine Serum Albumin
CMIA	Chemiluminescent Microparticle Immunoassay
Ct	Cycle threshold (qPCR read-out)
CVD	Cardiovascular Diseases
CXCL12	C-X-C Motif Chemokine Ligand 12
DAB	3,3′-Diaminobenzidine (chromogen)
DMEM	Dulbecco’s Modified Eagle Medium
FBS	Fetal Bovine Serum
HBSS	Hanks’ Balanced Salt Solution
H&E	Hematoxylin & Eosin (histology stain)
HIF-1α	Hypoxia-Inducible Factor 1-alpha
LAD/LDA	Left (Anterior) Descending Coronary Artery (rat ligation site)
MCP1	Monocyte Chemoattractant Protein 1
miRNA	MicroRNA (noncoding regulatory RNA)
MI	Myocardial Infarction
MZB	Marginal-Zone B (lymphocyte)
PBS	Phosphate-Buffered Saline
(RT)-qPCR	(Reverse-Transcription) Quantitative Polymerase Chain Reaction
RLU	Relative Light Units (chemiluminescence read-out)
ROS	Reactive Oxygen Species
SEM	Standard Error of the Mean
SPSS	Statistical Package for the Social Sciences (software)
TNF-α	Tumor Necrosis Factor alpha
TTC	2,3,5-Triphenyltetrazolium Chloride (infarct stain)
VEGF	Vascular Endothelial Growth Factor

## Appendix A

Histological evaluation includes changes in tissue architecture, fiber disorganization, epicardial and endocardial cellularity, myocyte integrity, nuclear morphology, interstitial characteristics, and neovascularization. Notable findings include the onset of inflammatory infiltrates, necrosis and loss of myocytes (72 h–7 d), fibrosis, and increased vascularity in damaged areas. The table summarizes qualitative histopathological observations across time points, demonstrating progressive inflammation, immune cell infiltration, and tissue remodeling postinjury. The percentage of cells as a semi-quantitative estimation is included, following the scores indicated in Table A2.

**Table A1:** Histological Evaluation.

Histology	Sham	24 h	72 h	7 Days	1 Month
Architecture	Preserve	Preserve	Altered and inflammatory infiltrate (3)	Altered by necrosis and inflammatory infiltrate (2)	Altered by fibrosis and inflammatory infiltrate (1)
Fiber disorganization	Absent	Absent	Absent	Absent, fibrosis (2)	Present, focal fibrosis (3)
Epicardium cellularity	Normal	Cellularity increase	High cellularity increase	Cellularity increase	Cellularity increase
Endocardium	Normal	Normal	Focal affectation	Focal affectation	Normal
Myocytes	Matures	Matures	Replaced by inflammatory infiltrate. Necrotic myocytes (3)	Replaced by inflammatory infiltrate: chronic hurt. Necrotic myocytes (2)	Replaced by inflammatory infiltrate and fibrosis.
Nuclei	Low variability	Low variability: hyper eosinophilia and pyknosis, focal	Disappeared in focal areas	Disappeared in focal areas	Disappeared in focal areas
Interstice	Normal	Normal/lightly cellularity increase	Inflammation: acute and chronic pain (3). Necrosis (4) and haemorrhage	Chronic inflammation (3) and necrotic myocytes (2)	Chronic inflammation (2)
New vascularity	No, normal	No, normal	Increased vessels (2) in inflammatory areas	Highly increased vessels (3) in inflammatory areas	Lightly increased vessels (1) in inflammatory areas

**Table A2:** Semi-quantitative estimation.

Feature	Scoring System (Semi-Quantitative)	Methodology/Markers	References
Necrosis	0 = none; 1 ≤ 25% of segment; 2 = 26–50%; 3 = 51–75%; 4 = 76–100% (transmurality per segment)	TTC staining (histology); LGE-CMR segmental hyperenhancement	[44,45]
Fibrosis	0 = none; 1 = mild; 2 = moderate; 3 = severe (based on % area or intensity)	Masson’s trichrome (histology); LGE-CMR (signal thresholding, e.g., >5 SD above remote myocardium)	[44,46]
Neovascularization	0 = none; 1 = rare; 2 = moderate; 3 = extensive (count per high-power field or area)	CD31/CD34 immunostaining (histology); not routinely quantified by CMR	[44]
Inflammation	0 = none; 1 = mild; 2 = moderate; 3 = severe (inflammatory cell count per high-power field)	H&E (histology); T2-weighted CMR (edema as surrogate for inflammation, >2 SD above remote myocardium)	[44]

Permanent occlusion (PO) was induced in Sprague Dawley rats by clamping the coronary descending aorta. Ischemic heart tissue was harvested at 24 h, 72 h, 7 days and 1 month. Then, immunohistochemistry, miRNAs determination, hematoxylin and eosin, Masson and TTC/Evans Blue staining were performed in paraffin-embedded sections. Additionally, serum samples from rats at 24 h, 72 h, 7 days and 1 month were also obtained for further determination.

Ethical and animal care considerations: Post-procedural animal welfare will be ensured through daily supervision and assessment of physical condition, feeding status, and wound care. The animal’s degree of suffering will be objectively evaluated using the Rat Grimace Scale and responsiveness checks, followed by analgesia administration to minimize surgical impact. To prevent complications, a validated surgical protocol is employed by expert personnel, including hygenicmeasures and precautions against pneumothorax (e.g., mechanical ventilation). Anesthetic complications such as hypothermia and cardiorespiratory depression will be closely monitored and prevented with a heat source and adjusted drug dosages followig veterinary guidance. Euthanasia will be performed as the humane endpoint if surgical complications—including massive hemorrhage, persistent fever, massive pneumothorax, or cardiogenic shock—arise and compromise the animal’s homeostasis.
